# Diet Quality Scores of Australian Adults Who Have Completed the Healthy Eating Quiz

**DOI:** 10.3390/nu9080880

**Published:** 2017-08-15

**Authors:** Rebecca L. Williams, Megan E. Rollo, Tracy Schumacher, Clare E. Collins

**Affiliations:** 1 Priority Research Centre for Physical Activity and Nutrition, University of Newcastle, Newcastle, NSW 2308, Australia; rebecca.williams@newcastle.edu.au (R.L.W.); megan.rollo@newcastle.edu.au (M.E.R.); 2 School of Health Sciences, Faculty of Health and Medicine, University of Newcastle, Newcastle, NSW 2308, Australia; tracy.schumacher@newcastle.edu.au; 3 University Department of Rural Health, University of Newcastle, Tamworth, NSW 2340, Australia

**Keywords:** diet variety, healthy eating quiz, Australian dietary intake, adults

## Abstract

Higher scores obtained using diet quality and variety indices are indicators of more optimal food and nutrient intakes and lower chronic disease risk. The aim of this paper is to describe the overall diet quality and variety in a sample of Australian adults who completed an online diet quality self-assessment tool, the Healthy Eating Quiz. The Healthy Eating Quiz takes approximately five minutes to complete online and computes user responses into a total diet quality score (out of a maximum of 73 points) and then categorizes them into the following groups: ‘needs work’ (<33), ‘getting there’ (33–38), ‘excellent’ (39–46), or ’outstanding’ (47+). There was a total of 93,252 first-time respondents, of which 76% were female. Over 80% of respondents were between 16–44 years of age. The mean total score was 34.1 ± 9.7 points. Females had a higher total score than males (*p* < 0.001) and vegetarians had higher total scores than non-vegetarians (*p* < 0.001). Healthy eating quiz scores were higher in those aged 45–75 years compared to 16–44 years (*p* < 0.001). When comparing Socioeconomic Indices for Areas deciles, those most disadvantaged had a lower total score than those least disadvantaged (*p* < 0.001). Repeat measures showed that those who scored lowest (needs work) in their first completion increased their total score by 3.2 ± 7.4 at their second completion (*p* < 0.001). While the Healthy Eating Quiz data indicates that individuals receiving feedback on how to improve their score can improve their diet quality, there is a need for further nutrition promotion interventions in Australian adults.

## 1. Introduction

Higher scores obtained using diet quality and variety indices are indicators of higher vitamin and mineral intakes and lower risk of chronic disease [1,2,3]. Despite a wealth of research that indicates dietary improvements can reduce the risk of lifestyle-related conditions such as obesity, type 2 diabetes, and cardiovascular disease [3], the prevalence of these conditions continues to rise [4]. This may indicate a need for scalable interventions that address common barriers such as access and time [5]. Diet quality indices are designed to be scored quickly and reflect the diversity within food groups. Food-based diet quality indices consider the variety of foods within food groups consumed in a given period and assign points based on diversity and/or frequency of intake. Across food-based diet quality indices, there is variation in how points are awarded and whether or not weightings are applied [6]. The Healthy Eating Quiz (HEQ) is based on the USA Recommended Food Score and does not apply weightings. We have previously shown that it is a valid estimate of usual nutrient intakes in population samples of toddlers, children, adolescents, and adults [1,2,7]. Higher scores reflect dietary patterns that align more closely with National Dietary Guidelines. To date, dietary assessment tools used in nutritional epidemiology have rarely been used as a public health nutrition assessment and education tool. Technology-based dietary assessment tools that automate dietary intake analysis and personalize feedback have the potential for broad population reach and significant reductions in the time associated with dietary assessment and brief intervention [8].

This short report aims to provide a brief overview of a publically available online diet quality and variety self-assessment index, the Healthy Eating Quiz (HEQ), and to describe the diet quality components and characteristics of Australians accessing the tool.

## 2. Materials and Methods

### 2.1. Healthy Eating Quiz

The online diet quality index, the HEQ was modelled on the Australian Recommended Food Score (ARFS) previously validated in adults [2], children and adolescents [7], and preschoolers [1], with the scoring algorithms transposed to system total and sub-scale scores in real-time [9]. The HEQ focuses on dietary variety within food groups recommended in the Australian Dietary Guidelines [10]. For example, the meat and alternatives food group encapsulates a range of differing foods, each with unique nutrient profiles i.e., red meat, fish, eggs, nuts, and legumes. The HEQ uses a sub-set of 70 questions from a validated food frequency questionnaire [11,12,13]. The overall score is comprised of eight sub-scales with 20 questions related to vegetables, 12 to fruit, 7 to meat/flesh foods, six to plant-based protein foods, 12 to breads and cereals, 10 to dairy foods, one to water, and two to spreads/sauces. Most foods are awarded one point for a consumption frequency of ≥once per week. Further detail on the scoring can be found elsewhere in Table 1 [7]. The HEQ score is calculated by summing the points for each item. The total score ranges from zero to 73. If respondents selected that they were a vegetarian (type not specified), they were assigned a score of zero for meat questions, while points for each of the vegetarian questions were doubled for those that were selected as consumed ‘at least once per week or more’ and one bonus point was awarded if both soybeans, tofu and other beans, lentils were scored as ‘at least once per week or more’. Soybeans and tofu, and other beans and lentils were awarded a bonus point as they have a more optimal nutrient profile within the vegetarian options category.

The HEQ takes approximately five minutes to complete online (www.healthyeatingquiz.com.au) and converts the user’s responses into total and sub-scale scores, which categorizes them into the following groups: ‘needs work’ (<33), ‘getting there’ (33–38), ‘excellent’ (39–46), or ’outstanding’ (47+). They also receive brief feedback on how to improve their dietary intake.

### 2.2. Participants

**Figure 1:**
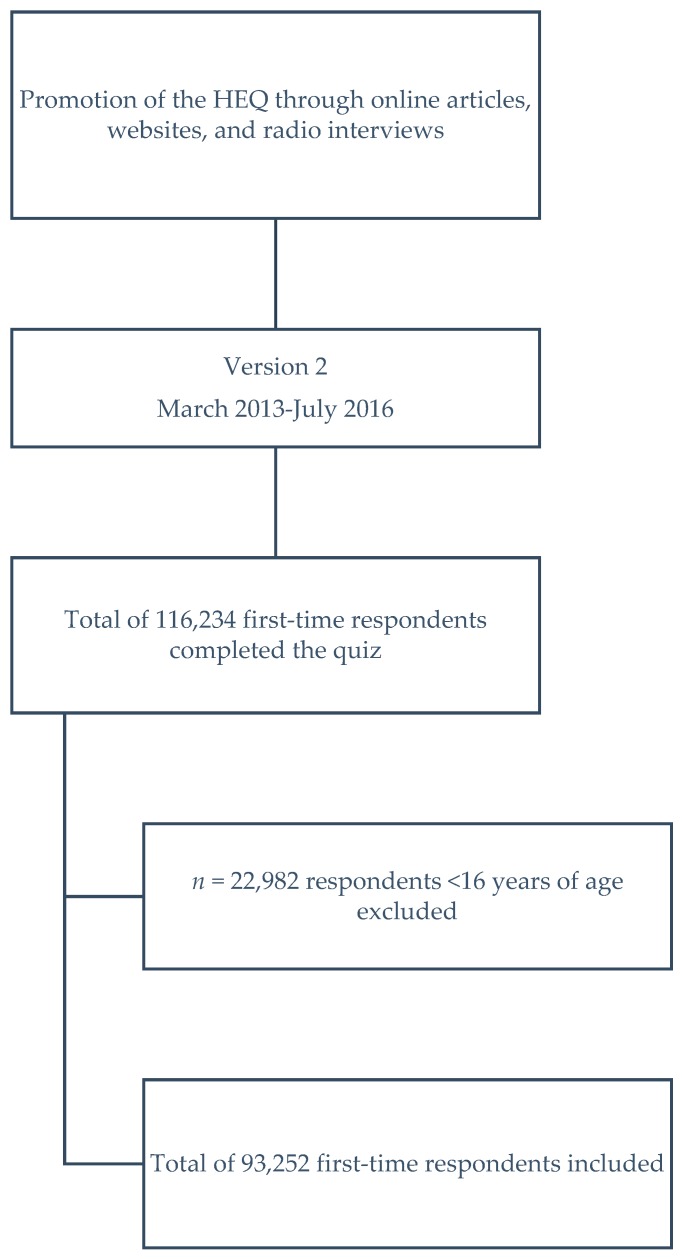
Flow chart of participant recruitment and selection.

The study sample comprises individuals who voluntary elect to complete the online HEQ, having located and accessed the HEQ web link either directly or from a website that includes a hyperlink to the HEQ (Figure 1). The web link to the HEQ was included by researchers in a number of online media articles about healthy eating including ‘The Conversation’ [14], and mentioned during radio interviews and national health promotion days such as Australia’s Healthy Weight Week [15]. The Healthy Eating Quiz was also listed on the website of the Dietitians Association of Australia [16], Back to Basics Cooking Club and Healthy Lifestyle Program [17], and Healthy Dads Healthy Kids [18].

### 2.3. Statistical Analysis

Data in the current analysis were obtained from HEQ respondents during March 2013–July 2016. All statistical analyses were conducted using Stata statistical software version 12.1 (StataCorp, College Station, TX, USA). All data were normally distributed; therefore, data are reported as means ± SD. An independent *t*-test was used to assess differences between sub-groups. Deciles of Socioeconomic Index for Areas (SEIFA) of advantage and disadvantage were input for those postcodes with four digits, representing Australian postcodes. A low SEIFA score indicates higher disadvantage and less advantage whereas a higher score indicates lower disadvantage and higher advantage. Data were also paired for respondents who completed the quiz more than once. If respondents completed the HEQ more than twice, data from the second completion was used and this had to be more than three weeks since initial completion to be included. Change in HEQ score was analyzed by baseline HEQ score category to examine the impact relative to baseline data. A paired *t*-test was used to assess changes within each category. A one-way analysis of covariance with Bonferroni correction was used to assess differences between categories. Multiple linear regression was also used to adjust potential confounding effects on relationships between HEQ total score and age (Model 1: *Y_HEQScore_* = *β*_0_ + *β*_1_*X_age_* + *β*_2_X_gender_ + *β*_3_X_SEIFA_ + e_i_), and HEQ total score and number of people meal shared with (Model 2: *Y_HEQScore_* = *β*_0_ + *β*_1_*X_sharedmeals_* + *β*_2_X_age_ + *β*_3_X_gender_ + *β*_4_X_SEIFA_ + e_i_). Confounding variables included in model 1 were gender and SEIFA, and in model 2, age, gender, and SEIFA. The level of significance was set at *p* < 0.05.

### 2.4. Ethics

Ethics approval was obtained from the University of Newcastle Human Research Ethics Committee (H-2016-0168). When completing the HEQ, respondents were given the option to consent for their de-identified data to be used by the University of Newcastle for research purposes. Only those selecting yes to this option are included in this report.

## 3. Results

Table 2 summarizes the scores for first-time respondents by age group and males and females separately. Table 3 displays scores for first-time respondents identifying themselves as vegetarians, while Table 4 summarizes scores by the number of people main meals are shared with and Australian SEIFA decile. Table 5 and Table 6 display HEQ scores by number of quiz completions and changes in HEQ score in paired data.

Of the 93,252 HEQ respondents, 76% were female. Over 80% of respondents were aged 16–44 years, with less than 1% over 75 years of age. Of the group, 9.8% reported they were vegetarians. However, only 6.8% of the total group were non-meat eating vegetarians (scored zero for unadjusted meat category), the remaining 3.0% reported eating meat, poultry, or fish.

The mean total HEQ score was 34.1 ± 9.7 out of 73 possible points (Table 2) which falls into the category of ‘Getting there’ in terms of overall diet quality. Respondents scored highest for variety within the vegetable subscale, reaching 55% of the maximum score. Fruit, meat, meat alternatives, and grains subscales reached between 43–48% of their maximum scores, with dairy reaching 40% of the maximum score. Vegetarians reached 63% of the maximum score for meat alternatives sub-scale.

### 3.1. Males Versus Females

Females scored higher than males for the vegetable, fruit, and meat alternative sub-scales as well as for total score (Total score: Females 34.5 ± 9.3; Males 33.1 ± 10.6; *p* < 0.001) (Table 2). Males scored higher for meat, grains, and dairy variety sub-scales.

### 3.2. Age Categories

Age categories were collapsed to evaluate differences in HEQ scores, indicating differences in diet quality, based on comparing younger versus older age groups. Those aged 16–44 years had higher variety with the grain sub-scale (*p* < 0.001) (Table 2), whereas those aged 45–75+ years reported greater vegetable, fruit, meat, meat alternative, dairy, and higher total variety scores (*p* < 0.001) (Table 2). However, analysis of age categories using a more narrow age range demonstrated that the age group of 75+ years had the lowest mean HEQ score of those aged 45 years and older, whereas those 16–24 years tended to have the lowest scores of all age groups, *p* < 0.001 (Table 2).

### 3.3. Vegetarians

Analyses comparing vegetarians and non-vegetarians compared adjusted HEQ scores, as described in the methods section. Vegetarians scored higher than non-vegetarians for the total HEQ score vegetable, fruit, adjusted meat alternatives, and grains subscales, *p* < 0.001 (Table 3). As expected, non-vegetarians scored higher for the unadjusted meat sub-scale and for the dairy sub-scale. Unadjusted data indicate that overall, vegetarians scored less than one point for the meat variety sub-scale, with 9% of vegetarians scoring three or more meat points (max score = 7). On average, vegetarians scored just above 3.5 points for meat alternatives, one point higher than non-vegetarians (*p* < 0.001).

### 3.4. Healthy Eating Quiz Score by Meal Sharing Characteristics

Those who consumed their main meals alone had significantly lower mean total HEQ scores compared to those who shared their main meals with one or two or more other people. There was also a significant difference between those who shared their main meals with one versus those sharing with two or more others, with the total HEQ score increasing in line with the number of people the main meals were shared with, *p* < 0.001 (Table 4).

### 3.5. SEIFA Deciles

There was a trend for increasing total HEQ scores as SEIFA deciles increased, with those classed in SEIFA 1 (most disadvantaged, least advantaged) having a mean total HEQ score of 33.3 ± 10.2 compared to a mean total HEQ score of 37.2 ± 8.6 (*p* < 0.001) in SEIFA 10 (Table 4). A trend for increasing HEQ scores in line with a higher SEIFA decile were also observed for the vegetable, fruit, meat alternative, and grain subscales, whereas no difference were observed for the meat and dairy subscales, *p* > 0.05.

### 3.6. Comparison by Completion Time

HEQ respondents who were not first-time completers of the quiz (2%) had significantly higher scores than first-time completers. They scored higher in all food sub-scales plus total HEQ score, with a mean of 4 ± 2 points higher for total score in repeat respondents versus first-time respondents (*p* < 0.001) (Table 5). When data were paired (Table 6), those who scored lower at baseline showed greater improvements in their total score than those who scored better at baseline. The ‘needs work’ category increased their total score by a mean of 3.2 ± 7.4, whereas those who were classed as ‘outstanding’ at baseline decreased scores by −3.0 ± 6.5 by their follow-up quiz.

### 3.7. Multiple Linear Regression

Results from the multiple linear regression indicate that there is a significant relationship between HEQ score and age when sex and SEIFA are accounted for in the model (*p* < 0.001, R^2^ = 0.031), with a one-unit increase in age category associated with a 0.60 point increase in HEQ score (coefficient = 0.60, 95% Confidence Interval [CI] 0.54–0.66). In the model investigating the relationship between HEQ score and number of people meals were shared with (age, sex, and SEIFA accounted for in the model), there was also a significant relationship (*p* < 0.001; R^2^ 0.0591) with an increase in category for the number of people meals were shared with associated with a 1.98 increase in HEQ score (coefficient 1.98, 95% CI: 1.84–2.12).

## 4. Discussion

The current study described the diet quality of Australian adults who completed the Healthy Eating Quiz, a brief online dietary assessment tool that evaluates overall diet quality and variety. Whilst females had greater overall HEQ scores, indicating higher overall variety with the core food groups, both males and females had relatively low total HEQ scores. This indicates that improvements can made in the variety of core foods within their diets, as a strategy to improve nutritional quality of population dietary intakes. This is important, as we have previously shown that higher total scores using this diet quality index indicates more optimal nutrient intake profiles [2,19].

There was a consistent trend towards older age groups (45 years+) having higher HEQ scores compared to younger age groups (16–44 years) and this was supported by multiple linear regression showing increases in HEQ score when sex and SEIFA were held constant. From 16 years of age, diet variety scores progressively increase, up until 75 years and above where a decrease in dietary variety was observed. The likelihood of eating alone increases with age, and it is not uncommon for individuals to prepare less wholesome meals when cooking and eating on their own [20,21]. Population data also show those over 65 years of age who live alone have the lowest weekly income of any life cycle, further increasing their likelihood of inadequate dietary intake [22]. As health status may also affect food choices and eating behaviors [23], the elderly may be at greater risk of poorer diet quality. It is important to note that while there were statistically significant differences in total score between age groups; the differences may not be clinically significant, meaning that they may not lead to differences in nutrient status. Therefore, findings highlight the importance of providing appropriate evaluation and support to optimize diet quality and nutrient intakes among all Australians.

Analysis of those identifying as vegetarian indicates that many still consume some meat (flesh) foods, suggesting that they were semi-vegetarian. Data from the Australian Longitudinal Study on Women’s Health (ALSWH) reported that 3% and 10% of the females in that cohort were vegetarians or semi-vegetarian, respectively [24]. Although the HEQ does not specifically ask the type of vegetarian, data in the current analysis suggests that there was a larger proportions of vegetarians compared to semi-vegetarians. This highlights that when conducting research in vegetarians, specifying the type of vegetarian should be reported, particularly if outcomes relate to intakes of nutrients found predominantly in flesh foods, such as haem iron and omega 3 fatty acids.

Findings from the unpaired analysis of the total sample indicate that respondents who completed the HEQ more than once had significantly higher HEQ scores. This suggests that Australian adults were able to use the brief personalized feedback provided in this initial assessment of their diet quality and variety and improve their HEQ score over time. However, when data were able to be paired, and categorized by their first time score, the change was driven by those with a lower score initially. The duration between first and second responses for the ‘needs work’ category was seven months. This timeframe is reasonable to expect that at least some of this change is a real increase in HEQ score. However, we also acknowledge that further evaluation of the utility of the HEQ in intervention studies is warranted.

One important limitation of the current analysis that needs to be acknowledged is that the sample from which the data are drawn, although large, may not be representative of the whole Australian population. It is likely to be influenced by bias, given those who completed the Healthy eating quiz chose to do so of their own accord. They therefore are likely to have a stronger interest in nutrition compared to those who have not completed the quiz. Additionally, as the HEQ score is modelled on foods representative of Australian foods, it may not be appropriate for use outside of Australia.

## 5. Conclusions

Analysis of data from Healthy Eating Quiz respondents highlights the need for further nutrition education and interventions to optimize diet quality in Australia. Data from the Healthy Eating Quiz do indicate that individuals completing the HEQ who receive brief personalized feedback on how to improve their scores are able to improve diet quality scores, especially for those with lower scores initially. Being an online format, the HEQ removes some common barriers to dietary intake assessment and feedback such as access and time, which increases potential reach to assess and educate Australian adults about nutrition more than has been possible previously.

## Figures and Tables

**Table 1:** Scoring method for items in the Healthy Eating Quiz.

Food Group	Items Giving 1 Point	Items Giving More Than 1 Point	ARFS *
Vegetables	3–4 nightly meals with vegetables; ≥1per week of each of the following vegetables: potato, pumpkin, sweet potato, cauliflower, green beans, spinach, cabbage or Brussels sprouts, peas, broccoli, carrots, zucchini or eggplant or squash, capsicum, corn, mushrooms, tomatoes, lettuce, celery or cucumber, avocado, onion or leek or shallots/spring onion.	2 points for ≥5 nightly meals with vegetables	21
Fruit	≥1 piece of fruit per day, ≥1 per week of each of the following fruit: canned fruit, fruit salad, dried fruit, apple or pear, orange or mandarin or grapefruit, banana, peach or nectarine or plum or apricot, mango or paw-paw, pineapple, grapes or strawberries or blueberries, melon (any variety).		12
Protein foods- Meat/flesh	≤1 serve of minced meat per month but greater than never; 1–4 serve per week of: beef or lamb with or without sauce and/or vegetables per week chicken without batter or crumbing but with or without sauce and/or vegetables, pork with or without sauce and/or vegetables; ≥1 per week of fresh fish, canned tuna or salmon or sardines, other seafood (e.g., prawns, lobster).		7
Vegetarian sources of protein	≥1 per week of the following: nuts (e.g., peanuts, almonds), nut butters, eggs, soybeans or tofu, baked beans, other beans or lentils (e.g., chickpeas, split peas).		6
Breads and cereals	Usual bread choice is ‘other’ (e.g., rye, high-fiber white); ≥1 per week of the following: muesli, cooked porridge, breakfast cereal (e.g., Weet-bix, Nutri-grain, Cornflakes), bread or pita bread or toast, English muffin or bagel or crumpet, rice, other grains (e.g., couscous, burghul), noodles (e.g., egg noodles, rice noodles), pasta, tacos or burritos or enchiladas, clear soup with rice or noodles.	2 points if usual bread choice is ‘brown’ (multigrain or wholemeal).	13
Dairy	≥2 serves of: milk, yoghurt or cheese per day; ≥1 serve per week but ≤1 serves per day of flavoured milk, ice cream, frozen yoghurt; ≥1 serve per week but ≤4 serves per day of cheese, cheese spread or cream cheese; ≥1 serve per week of plain milk, yoghurt (not frozen), cottage cheese or ricotta.	2 points if usual type of milk is reduced fat milk or skim milk, or soy milk	11
Water	≥4 glasses of water (including tap, unflavoured bottled water, and unflavoured mineral water).		1
Spreads/ sauces	≥1 serve per week of: yeast extract spread; tomato or barbecue sauce		2
**Total**			**73**

* ARFS = Australian Recommended Food Score.

**Table 2:** Total and sub-category Healthy Eating Quiz scores (means ± S.D.) for first-time respondents (*n* = 93,252).

	Total Score	Vegetables	Fruit	Meat *	Meat Alt. *	Grains	Dairy
**Age Groups**							
Total (*n* = 93,252)	**34.1 ± 9.7**	**11.5 ± 4.3**	**5.5 ± 2.9**	**3.0 ± 1.6**	**2.6 ± 1.3**	**5.7 ± 2.3**	**4.0 ± 2.1**
16–24 years (*n* = 37,229)	32.6 ± 9.7	10.6 ± 4.3	5.4 ± 2.8	2.7 ± 1.6	2.5 ± 1.4	5.6 ± 2.3	3.9 ± 2.1
25–34 years (*n* = 26,068)	33.1 ± 8.9	11.5 ± 4.0	4.8 ± 2.6	2.9 ± 1.6	2.6 ± 1.3	5.5 ± 2.3	3.8 ± 2.0
35–44 years (*n* = 12,322)	34.5 ± 9.1	12.3 ± 4.0	5.1 ± 2.8	3.2 ± 1.6	2.7 ± 1.3	5.6 ± 2.4	3.8 ± 2.0
45–54 years (*n* = 9214)	35.3 ± 8.9	12.9 ± 3.8	5.4 ± 2.8	3.4 ± 1.6	2.6 ± 1.3	5.4 ± 2.3	3.8 ± 2.0
55–64 years (*n* = 5840)	36.5 ± 8.7	13.5 ± 3.6	5.8 ± 2.7	3.5 ± 1.6	2.7 ± 1.2	5.4 ± 2.2	4.0 ± 1.9
65–74 years (*n* = 2111)	37.2 ± 8.5	13.7 ± 3.6	6.1 ± 2.6	3.6 ± 1.6	2.7 ± 1.2	5.3 ± 2.1	4.2 ± 1.9
75+ years (*n* = 468)	35.8 ± 15.3	12.1 ± 6.0	6.2 ± 3.7	3.2 ± 1.8	2.9 ± 1.8	5.7 ± 3.2	4.0 ± 2.3
16–44 years (*n* = 75,619)	33.1 ± 9.4	11.2 ± 4.2	5.1 ± 2.8	2.9 ± 1.6	2.6 ± 1.3	5.6 ± 2.3	3.8 ± 2.1
45–75 years+ (*n* = 17,633)	35.9 ± 9.0	13.2 ± 3.8	5.6 ± 2.8	3.5 ± 1.6	2.7 ± 1.3	5.4 ± 2.3	3.9 ± 2.0
*p*-value	<0.001	<0.001	<0.001	<0.001	<0.001	<0.001	<0.001
16–44 years vs. 45–75+ years							
**Sex**							
Females (*n* = 71,290)	**34.5 ± 9.3**	**11.8 ± 4.2**	**5.7 ± 2.8**	**3.0 ± 1.6**	**2.6 ± 1.3**	**5.7 ± 2.3**	**3.9 ± 2.1**
16–24 years (*n* = 27,977)	33.0 ± 9.4	10.9 ± 4.2	5.5 ± 2.8	2.6 ± 1.6	2.5 ± 1.4	5.6 ± 2.2	3.8 ± 2.1
25–34 years (*n* = 20,033)	33.8 ± 8.6	11.8 ± 3.9	5.0 ± 2.6	2.9 ± 1.6	2.7 ± 1.3	5.6 ± 2.2	3.8 ± 2.0
35–44 years (*n* = 9516)	35.1 ± 8.9	12.6 ± 3.9	5.3 ± 2.7	3.2 ± 1.6	2.7 ± 1.3	5.6 ± 2.3	3.9 ± 2.0
45–54 years (*n* = 7393)	35.8 ± 8.9	13.2 ± 3.7	5.5 ± 2.8	3.4 ± 1.6	2.7 ± 1.3	5.4 ± 2.3	3.9 ± 2.0
55–64 years (*n* = 4544)	37.0 ± 8.6	13.8 ± 3.5	5.8 ± 2.7	3.5 ± 1.6	2.7 ± 1.2	5.4 ± 2.2	4.1 ± 2.0
65–74 years (*n* = 1556)	37.9 ± 8.4	14.1 ± 3.5	6.3 ± 2.5	3.7 ± 1.6	2.7 ± 1.2	5.3 ± 2.1	4.3 ± 1.9
75+ years (*n* = 271)	35.1 ± 14.2	11.8 ± 5.8	6.1 ± 3.5	3.1 ± 1.8	2.8 ± 1.6	5.4 ± 3.2	4.1 ± 2.3
Males (*n* = 21,962)	**33.1 ± 10.6**	**10.7 ± 4.5**	**5.2 ± 3.1**	**3.2 ± 1.6**	**2.5 ± 1.4**	**5.7 ± 2.5**	**4.0 ± 2.1**
16–24 years (*n* = 9252)	31.5 ± 10.5	9.7 ± 4.5	5.0 ± 3.0	3.0 ± 1.6	2.4 ± 1.4	5.6 ± 2.3	4.0 ± 2.1
25–34 years (*n* = 6035)	30.7 ± 9.3	10.4 ± 4.3	4.2 ± 2.7	3.0 ± 1.6	2.5 ± 1.3	5.3 ± 2.3	3.5 ± 2.0
35–44 years (*n* = 2806)	32.6 ± 9.4	11.4 ± 4.2	4.5 ± 2.8	3.3 ± 1.6	2.6 ± 1.3	5.4 ± 2.4	3.6 ± 1.9
45–54 years (*n* = 1821)	33.4 ± 9.0	11.9 ± 4.1	4.9 ± 2.8	3.4 ± 1.6	2.6 ± 1.3	5.4 ± 2.3	3.6 ± 2.0
55–64 years (*n* = 1296)	34.7 ± 8.7	12.6 ± 4.0	5.2 ± 2.7	3.5 ± 1.6	2.7 ± 1.2	5.4 ± 2.2	3.7 ± 1.9
65–74 years (*n* = 555)	35.1 ± 8.2	12.6 ± 3.6	5.7 ± 2.6	3.6 ± 1.6	2.6 ± 1.2	5.3 ± 2.1	4.0 ± 2.0
75+ years (*n* = 197)	36.8 ± 16.6	12.5 ± 6.2	6.3 ± 3.9	3.3 ± 1.8	3.0 ± 1.9	6.0 ± 3.5	4.0 ± 2.2
*p*-value	<0.001	<0.001	<0.001	<0.001	<0.001	<0.001	<0.001
females vs. males							

* Unadjusted meat and meat alternative score; *^#^* Comparing total scores only.

**Table 3:** Total and sub-category Healthy Eating Quiz scores (means ± S.D.) for vegetarians.

	Total Score	Vegetables	Fruit	Meat	Meat Alt.	Meat Adj. *	Meat Alt. Adj. *	Grains	Dairy
Vegetarian									
Total (*n* = 9093)	37.3 ± 9.9	12.6 ± 4.1	6.1 ± 2.8	0.7 ± 1.2	3.6 ± 1.4	0 ± 0	7.6 ± 3.1	6.2 ± 2.4	3.3 ± 2.3
16–24 years (*n* = 4470)	36.5 ± 9.5	12.2 ± 4.0	6.0 ± 2.7	0.6 ± 1.1	3.5 ± 1.4	0 ± 0	7.5 ± 3.1	6.1 ± 2.3	3.1 ± 2.3
25–34 years (*n* = 2556)	37.0 ± 8.7	12.8 ± 3.7	5.3 ± 2.6	0.6 ± 1.0	3.7 ± 1.3	0 ± 0	8.0 ± 2.9	6.0 ± 2.3	3.2 ± 2.2
35–44 years (*n* = 922)	38.1 ± 9.3	13.3 ± 3.9	5.7 ± 2.7	0.7 ± 1.1	3.7 ± 1.3	0 ± 0	8.0 ± 2.9	6.2 ± 2.4	3.4 ± 2.2
45–54 years (*n* = 599)	39.0 ± 8.9	14.0 ± 3.7	6.2 ± 2.7	0.8 ± 1.2	3.7 ± 1.3	0 ± 0	8.1 ± 2.9	5.9 ± 2.4	3.2 ± 2.3
55–64 years (*n* = 365)	39.5 ± 9.5	14.1 ± 3.9	6.5 ± 2.6	0.8 ± 1.2	3.8 ± 1.4	0 ± 0	8.1 ± 3.1	5.9 ± 2.1	3.5 ± 2.2
65–74 years (*n* = 108)	38.9 ± 9.2	14.49 ± 3.8	6.4 ± 2.4	1.1 ± 1.3	3.7 ± 1.3	0 ± 0	8.0 ± 3.0	5.2 ± 2.2	3.6 ± 2.0
75+ years (*n* = 73)	35.5 ± 24.0	11.0 ± 8.6	6.5 ± 5.0	1.8 ± 1.7	3.5 ± 2.4	0 ± 0	7.4 ± 5.3	5.9 ± 4.9	3.2 ± 2.3
Non-vegetarian									
Total (*n* = 84,159)	33.8 ± 9.6	11.4 ± 4.3	5.5 ± 2.9	3.3 ± 1.5	2.5 ± 1.3	3.3 ± 1.5	2.5 ± 1.3	5.7 ± 2.3	4.0 ± 2.0
16–24 years (*n* = 32,759)	32.1 ± 9.6	10.4 ± 4.3	5.3 ± 2.8	3.0 ± 1.5	2.3 ± 1.3	3.0 ± 1.5	2.3 ± 1.3	5.5 ± 2.2	4.0 ± 2.1
25–34 years (*n* = 23,512)	32.7 ± 8.8	11.4 ± 4.0	4.8 ± 2.6	3.2 ± 1.5	2.5 ± 1.3	3.2 ± 1.5	2.5 ± 1.3	5.5 ± 2.2	3.8 ± 2.0
35–44 years (*n* = 11,400)	34.2 ± 9.0	12.2 ± 4.0	5.1 ± 2.8	3.4 ± 1.4	2.6 ± 1.3	3.4 ± 1.4	2.6 ± 1.3	5.5 ± 2.3	3.8 ± 2.0
45–54 years (*n* = 8615)	35.0 ± 9.0	12.9 ± 3.8	5.3 ± 2.8	3.6 ± 1.4	2.6 ± 1.2	3.6 ± 1.4	2.6 ± 1.2	5.4 ± 2.3	3.8 ± 1.9
55–64 years (*n* = 5475)	36.3 ± 8.6	13.5 ± 3.6	5.8 ± 2.7	3.7 ± 1.4	2.7 ± 1.2	3.7 ± 1.4	2.7 ± 1.2	5.3 ± 2.2	4.0 ± 1.9
65–74 years (*n* = 2003)	37.1 ± 8.4	13.7 ± 3.6	6.1 ± 2.6	3.8 ± 1.4	2.6 ± 1.2	3.8 ± 1.4	2.6 ± 1.2	5.3 ± 2.1	4.3 ± 1.9
75+ years (*n* = 395)	35.8 ± 13.1	12.3 ± 5.3	6.1 ± 3.4	3.5 ± 1.7	2.8 ± 1.6	3.5 ± 1.7	2.8 ± 1.6	5.6 ± 2.9	4.2 ± 2.2
*p*-value	<0.001	<0.001	<0.001	<0.001	<0.001	<0.001	<0.001	<0.001	<0.001
vegetarian vs non-vegetarian ^#^									

* Adjusted score for vegetarians: Respondents answering yes to vegetarian received zero for meat intake and additional points for meat alternatives; *^#^* Comparing total scores only.

**Table 4:** Total and sub-category scores (means ± S.D.) for other sub-groups.

	Total Score	Vegetables	Fruit	Meat *	Meat Alt. *	Grains	Dairy
**Number of people meals are shared with**							
Yourself (*n* = 33,101)	31.0 ± 9.9	10.1 ± 4.4	5.1 ± 2.9	2.6 ± 1.6	2.5 ± 1.4	5.2 ± 2.3	3.7 ± 2.1
1 other person (*n* = 35,313)	34.5 ± 8.8	12.1 ± 4.0	5.2 ± 2.7	3.1 ± 1.6	2.6 ± 1.3	5.7 ± 2.2	3.9 ± 2.0
≥2 other people (*n* = 44,004)	36.2 ± 9.5	12.1 ± 4.1	6.1 ± 2.9	3.3 ± 1.6	2.5 ± 1.4	6.1 ± 2.3	4.2 ± 2.1
*p*-value	<0.001	<0.001	<0.001	<0.001	>0.050	<0.001	<0.001
yourself vs. ≥ 2 other people ^#^							
*p*-value	<0.001	<0.001	<0.001	<0.001	<0.001	<0.001	<0.001
1 other person vs. ≥ 2 other people ^#^							
SEIFA decile ^†^							
1 (*n* = 967)	33.3 ± 10.2	12.0 ± 4.3	4.9 ± 3.0	3.2 ± 1.6	2.3 ± 1.4	5.3 ± 2.3	3.8 ± 2.0
2 (*n* = 1356)	34.6 ± 10.0	12.5 ± 4.3	5.4 ± 2.9	3.4 ± 1.5	2.3 ± 1.3	5.4 ± 2.3	3.9 ± 2.0
3 (*n* = 1743)	35.4 ± 9.6	12.9 ± 4.1	5.5 ± 2.9	3.3 ± 1.6	2.4 ± 1.4	5.5 ± 2.3	4.0 ± 2.0
4 (*n* = 2270)	35.2 ± 9.5	12.9 ± 4.1	5.3 ± 2.9	3.4 ± 1.6	2.4 ± 1.3	5.5 ± 2.3	3.9 ± 2.0
5 (*n* = 2362)	35.5 ± 9.4	13.0 ± 4.0	5.3 ± 2.8	3.4 ± 1.6	2.4 ± 1.3	5.7 ± 2.3	3.9 ± 2.0
6 (*n* = 3397)	35.8 ± 9.1	13.0 ± 4.0	5.4 ± 2.8	3.3 ± 1.5	2.5 ± 1.3	5.7 ± 2.4	4.0 ± 2.0
7 (*n* = 2640)	35.4 ± 8.8	13.1 ± 3.8	5.3 ± 2.7	3.3 ± 1.5	2.5 ± 1.3	5.5 ± 2.2	3.8 ± 1.9
8 (*n* = 3551)	36.3 ± 8.9	13.3 ± 3.7	5.5 ± 2.7	3.3 ± 1.6	2.6 ± 1.3	5.8 ± 2.2	4.0 ± 1.9
9 (*n* = 5212)	36.9 ± 8.5	13.4 ± 3.6	5.6 ± 2.7	3.4 ± 1.6	2.7 ± 1.3	5.9 ± 2.2	4.0 ± 1.9
10 (*n* = 5548)	37.2 ± 8.6	13.5 ± 3.6	5.7 ± 2.7	3.4 ± 1.6	2.7 ± 1.3	5.9 ± 2.2	4.0 ± 2.0
*p*-value SEIFA 1 vs. SEIFA 10 ^#^	<0.001	<0.001	<0.001	>0.050	<0.001	<0.001	<0.01

* Unadjusted score for meat and meat alternatives; ^#^ Comparing total scores only; ^†^ SEIFA = Socio-economic Index for Areas 1 = most disadvantaged and least advantaged, 10 = most advantaged and least disadvantaged.

**Table 5:** Comparison of HEQ score between first-time and repeat respondents (un-paired data).

	Total Score	Vegetables	Fruit	Meat *	Meat Alt. *	Grains	Dairy
**Number of times completed quiz *^#^***							
First time (*n* = 116,234)	34.1 ± 9.7	11.5 ± 4.3	5.5 ± 2.9	3.0 ± 1.6	2.6 ± 1.3	5.7 ± 2.3	4.0 ± 2.1
Repeat (*n* = 3739)	37.9 ± 11.6	12.4 ± 4.8	6.5 ± 3.0	3.1 ± 1.8	2.9 ± 1.5	6.4 ± 2.6	4.4 ± 2.1
*p*-value first time vs. repeat ^†^	<0.001	<0.001	<0.001	<0.001	<0.001	<0.001	<0.001

* Unadjusted score for meat and meat alternatives; ^#^ Un-paired data; ^†^ Comparing total scores only.

**Table 6:** Comparison of change in total HEQ score by baseline classification (paired data *n* = 1044).

Change in Total Score by Baseline Category *	Total Score ^#^	95% CI ^‡^	*p*-Value ^†^	Mean Time between HEQ Completions (Months)
Needs work (*n* = 303)	3.1 ± 7.3 ^b,c,d^	−3.9, −2.4	***p* < 0.001**	7.0
Getting there (*n* = 204)	0.5 ± 6.7 ^a,d^	−1.3, 0.4	*p* = 0.27	9.9
Excellent (*n* = 280)	−0.4 ± 6.0 ^a,d^	−0.3, 1.0	*p* = 0.28	10.0
Outstanding (*n* = 97)	−3.5 ± 7.1 ^a,b,c^	2.2, 4.8	***p* < 0.001**	10.6

* Paired data only; ^#^ Change over time; ^†^ Significant change over time; ^‡^ CI = Confidence Interval; ^a^ Significantly different from ‘needs work’; ^b^ Significantly different from ‘getting there’; ^c^ Significantly different from ‘excellent’; ^d^ Significantly different from ‘outstanding’.
